# Determination of true ratios of different *N*-glycan structures in electrospray ionization mass spectrometry

**DOI:** 10.1007/s00216-017-0235-8

**Published:** 2017-03-07

**Authors:** Clemens Grünwald-Gruber, Andreas Thader, Daniel Maresch, Thomas Dalik, Friedrich Altmann

**Affiliations:** Department of Chemistry, University of Natural Resources and Life Sciences, Vienna, Muthgasse 18, 1190 Vienna, Austria

**Keywords:** *N*-Glycan, Sialylation, Quantitative glycomics, Mass spectrometry, Electrospray ionization

## Abstract

An ideal method for the analysis of *N*-glycans would both identify the isomeric structure and deliver a true picture of the relative, if not absolute, amounts of the various structures in one sample. Porous graphitic carbon chromatography coupled with electrospray ionization mass spectrometry (ESI-MS) detection has emerged as a method with a particularly high potential of resolving isomeric oligosaccharides, but little attention has so far been paid to quantitation of the results obtained. In this work, we isolated a range of structures from Man5 to complex type *N*-glycans with zero to four sialic acids and blended them into an equimolar “glyco tune mix”. When subjected to liquid chromatography–ESI-MS in positive and negative modes, the glyco tune mix clearly demonstrated the futility of quantitation of *N*-glycans of different overall composition, different number of sialic acids, and strongly differing size without compensation for their very different molar responses. Relative quantitation of human plasma *N*-glycans was performed with correction factors deduced from this external glyco tune mix. Addition of just one isotope-coded internal standard with enzymatically added ^13^C-galactose led to absolute quantification in the same experiment.

**Electronic supplementary material:**

The online version of this article (doi:10.1007/s00216-017-0235-8) contains supplementary material, which is available to authorized users.

**Graphical Abstract:**
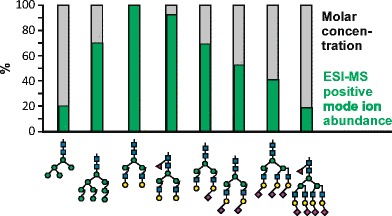
Discrepancy between desirable (grey bars) and real (green bars) relative ion abundance of equimolar amounts of glycans in positive mode ESI-MS.

## Introduction

Structural assignment of *N*-glycans and *O*-glycans has been a major issue in the last few decades, with the clearest advances having been achieved with mass spectrometry (MS) [1] and even more with liquid chromatography (LC)–MS [2, 3]. In terms of power to define a particular structure, MS surpasses chromatographic techniques such as hydrophilic interaction LC (HILIC)–high-performance LC (HPLC) or capillary zone electrophoresis of fluorescently labeled glycans [1]. These techniques, however, offer the advantage of an inherently identical molar response of all *N*-glycan species in a sample because of the invariable stoichiometry of the fluorophore [4, 5]. Correct relative quantitation of the components within a sample therefore requires well-separated peaks as facilitated by the latest ultraperformance HILIC columns [6]. Even then, with samples being more complex than antibody or plasma *N*-glycans, peak overlap will increasingly become a problem and requires the higher definition power of MS.

The term “relative quantitation” may be understood in two rather different ways. First, it refers to the comparison of two or more samples with the aim of finding upregulated or downregulated glycoforms. Second, it can mean determination of the correct ratios of different glycan structures within one sample.

The first aim, comparison of different samples, can be accomplished “label-free” by consecutive analyses [7–9] or it can be realized by derivatization with isotopically labeled reagents. Light and heavy forms of 2-aminopyridine [10], anthranilic acid [11, 12], 2-aminobenzoic acid [13, 14], aniline [15–17], and phenyl-3-methyl-5-pyrazolone [18, 19] have been used. Other chemistries targeting the reducing end used carbonyl-reactive tandem mass tags [20], Girard’s reagent [21], TMT-labeling of *N*-glycosylamines or reducing glycans [22, 23], or hydrazide based reagents [24, 25]. Two samples from cell cultures can be compared by metabolic incorporation of ^15^N into *N*-acetylglucosamine (GlcNAc) residues [26]. Several groups used permethylation to introduce heavy isotopes [27–29], whereby H/D permethylation introduced a remarkably strong deuterium effect [29]. Only a few of these approaches have been applied to biologicals samples such as glycans from carcinoma cell lines [20, 26], differentiating murine stem cells [28], or serum from healthy donors [16] and from patients with esophageal diseases [23, 29]. One study screened ovarian cancer samples from a biorepository and identified a number of glycans.

However, none of these approaches consider the differing molar response of glycans of different size, different number of sialic acids, different general architecture (oligomannosidic vs complex type), different charge states, and different tendency to form adducts with sodium or ammonium in electrospray ionization (ESI) MS [30]. In addition, chemical modification of glycans is essentially not compatible with the one method that has the greatest ability to separate structural isomers; that is, porous graphitic carbon (PGC) chromatography coupled with ESI-MS [2, 3]. Nevertheless, impressive separation of permethylated glycans by PGC chromatography has been reported recently [31], and allows the introduction of methyl groups of differing isotopic composition. However, PGC separation of reduced but otherwise unmodified glycans is still the standard format [3].

A solution to the problem is the use of internal standards labeled without alteration of the overall structure, which has recently been presented in the form of ^13^C-labeled N-acetylated glycans [30]. A range of glycan structures covering the species relevant for the analysis of monoclonal antibodies was prepared, including a disialylated glycan. Many glycoproteins, however, contain trisialylated and tetrasialylated *N*-glycans, and in our experience and that of others these more complex glycans show the strongest deviations of molar response [32–34]. Preparation of isotopically labeled glycans of this complexity, however, becomes a highly demanding task, and hence the resulting standard mixtures would be too expensive for routine use. Therefore, Mehta et al. [35] concentrated on a set of three natural glycans with zero or two sialic acids for the analysis of permethylated glycans by nanospray MS and matrix-assisted laser desorption ionization time-of-flight (MALDI-TOF) MS [35]. Both strategies yield absolute quantitation and hence also true relative proportions of the glycans considered. For characterization of biopharmaceutical glycoproteins, relative quantitation appears to be the more relevant task, which is particularly compounded by multiantennary, highly sialylated structures if it is conducted by MS [34]. A recent approach to this task therefore involved enzymatic simplification of *N*-glycans by sialidase, galactosidase, and fucosidase. Thereby, the original bias toward high-mannose structures was clearly diminished [34]. MALDI-TOF MS of permethylated glycans circumvents the problems arising from carboxyl groups and is held to reflect truly the molar proportions of the glycans in a sample. This was convincingly substantiated in a recent study by comparison of MALDI-TOF MS and HILIC–HPLC data [36].

In this work we generated an equimolar mixture of *N*-glycans with natural isotope patterns covering a wide range of structures from Man5 to tetrasialylated glycans (Table 1). This calibration mixture clearly demonstrated the very different molar responses of different glycan species and the usefulness of this standard for instrument tuning and determination of true molar ratios. Absolute quantitation—if required—can then be achieved with application of just one or maybe two isotopically labeled internal standards, which in this work were obtained via enzymatically prepared UDP-^13^ *C*_6_-galactose.

**Table 1:**
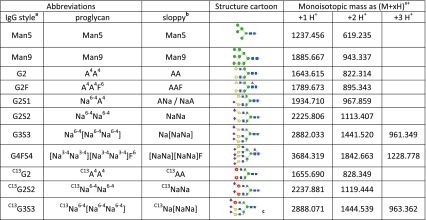
*N*-Glycan structures used in this work with their names and masses used. The IgG style abbreviations give the number of galactoses (*G*) and sialic acids (*S*) and presence of fucose (*F*)

The ProGlycAn nomenclature gives a complete description of the structure starting with the terminal residues on the 6-arm (see http://www.proglycan.com)

^a^As, for example, used at http://prozyme.com/collections/n-glycans-standards

^b^Without linkage superscripts

^c^Location of the ^13^C-galactose not defined

## Materials and methods

### Preparation of complex *N*-glycan standards

The nonsialylated diantennary glycan A^4^A^4^ (G2) was pre-pared from a pepsin digest of bovine fibrin by digestion with PNGase A and reduction with NaBH_4_. Final purification was achieved by chromatography on PGC on a 3 mm x 150 mm column (Thermo Scientific, Vienna, Austria) with a gradient from 5 to 30% acetonitrile in 65 mM ammonium formate of pH 3.0. Fractions were analyzed by capillary PGC-LC–ESI-MS [33].

The fucosylated standard A^4^A^4^F^6^ (“AAF”; G2F; see Table 1 for structures and their abbreviations) was isolated by preparative PGC chromatography from human IgG [32].

The monosialyated and disialyated glycans Na^6-4^A^4^/A^4^Na^6-4^ (G2S1) and Na^6-4^Na^6-4^ (“NaNa”; G2S2) were obtained by incubation of G2 (A^4^A^4^) with α2,6-sialyltransferase. To this end, a His-tagged version of rat ST6Gal I devoid of 96 residues at the N-terminus was expressed in the baculovirus/insect cell system. The G2S1 isomers (Na^6-4^A^4^ and A^4^Na^6-4^) and G2S2 were isolated by preparative PGC.

The trisialylated standard Na^6-4^[Na^6-4^ Na^6-4^] (G3S3) was isolated from the glycans released from bovine fetuin [37]. The tetrasialylated [Na^3-4^ Na^3-4^][Na^3-4^ Na^3-4^]F^6^ (G4FS4) was extracted by preparative PGC chromatography from erythropoietin, which was obtained as a by-product of a feasibility study for biosimilar production.

Man5 was prepared from *Aspergillus oryzae* amylase by PGC chromatography. Man9 was isolated from the *N*-glycan pool of white beans by HILIC on a TSKgel Amide-80 column (Tosoh Bioscience, Griesheim, Germany) [38].

The reference compounds were dried several times to remove any ammonium acetate or formate, taken up in water, and subjected to amino sugar analysis with consideration of the molar content of amino sugar [39].

### Preparation of high-mannose *N*-glycan standards

Fungal amylase was purified by passage over Sepharose S100 with 50 mM ammonium acetate of pH 6.0. A pepsin digest of the enzyme was passed over Sephadex G50m with 1% acetic acid as the solvent. The glycopeptide fraction was then treated with peptide *N*-glycosidase A. The digest was passed through a C_18_ silica cartridge (HyperSep C_18_, 25 mg; Thermo Scientific, Waltham MA, USA) and the flow-through was applied to a PGC cartridge (HyperSep Hypercarb, 25 mg; Thermo Scientific). *N*-Glycans were eluted with 50% acetonitrile in ammonium formate buffer of pH 3.0. As this material contained about 60% Man6, it was digested with recombinant α-1,2-specific mannosidase MNS1 from *Arabidopsis thaliana* [38]. The digest was again subjected to the purification steps described above. The purity of the final product was verified by PGC-LC–ESI-MS [40].

### Preparation of ^13^C_6_-labeled complex *N*-glycans

UDP-^13^ *C*_6_-galactose was prepared by incubation of ^13^ *C*_6_-galactose (Cambridge Isotope Laboratories, Tewksbury, MA, USA) with galactokinase (Sigma-Aldrich, Vienna, Austria). The galactose 1-phosphate was converted to the nucleotide sugar in the presence of UPD-glucose by human galactose 1-phosphate uridylyltransferase, which was recombinantly expressed in *Escherichia coli* BL21 and purified via its His_6_-tag (a yeast enzyme with this activity is now commercially available from Sigma-Aldrich ). The UDP-^13^ *C*_6_-galactose was finally purified by PGC chromatography with use of a slightly alkaline buffer as described in [41].

This UDP-^13^ *C*_6_-galactose was then used to regalactosylate fibrin glycans previously degalactosylated by *Aspergillus oryzae* β-galactosidase [33]. Bovine β-1,4-galactosyltransferase (Sigma-Aldrich, Vienna, Austria) was used in 50 mM tris(hydroxymethyl)aminomethane–HCl buffer for transfer of ^13^ *C*_6_-galactose. The thus produced ^C13^A^4^A^4^ (^C13^G2) was sialylated with α-2,6-sialyltransferase (see earlier) to arrive at singly and doubly sialylated (^C13^G2S2) standards with a 12-Da increment compared with the natural versions.

A trisialylated standard was prepared by partial degalactosylation of fetuin asialo *N*-glycans, isolation of the G2 form, followed by incorporation of just one ^13^ *C*_6_-galactose and α-2,6-sialylation.

### Quantification of individual *N*-glycans

The isolated standard compounds, both natural and heavy isotopologues, were subjected to hydrolysis with 4 M HCl acid for 4 h at 100 °C followed by reduction with NaBH_4_ and amino sugar analysis [39]. Analyses were repeated at least four times. Relative standard deviations of better than 5.5% were obtained. Molar concentrations were calculated on the basis of the respective glycan structure under the assumption of near to complete recovery of GlcNAc residues as glucosaminitols.

### Quantitative *N*-glycan analysis of serotransferrin, human plasma, and human serum albumin

Transferrin, human serum albumin (HSA), and human plasma (obtained from buffy coat preparations purchased from the University Clinic for Blood Group Serology and Transfusion Medicine, Graz, Austria) were digested with PNGase F as described in [8]. In short, the sample was denatured in 2% sodium dodecyl sulfate at 60 °C for 10 min. On dilution with 100 mM ammonium bicarbonate buffer containing 2% Igepal CA-630 (Sigma-Aldrich, Vienna, Austria) PNGase F (0.4 mU per 6 μL plasma; Roche, Mannheim, Germany) was added. The mixture was incubated for 16 h at 37 °C, whereupon 151 pmol of the isotope-coded ^C13^G2S2 standard per microliter of plasma was added to the sample. Released glycans were purified with PGC solid-phase extraction cartridges (HyperSep C_18_, 25 mg; Thermo Scientific, Waltham, MA, USA). Elution was done with 55% acetonitrile in ammonium formate buffer. Finally, the glycans were reconstituted in high-quality water. All experiments were done in triplicate.

### MS measurement and data interpretation

All samples were measured in positive and negative mode with two different instruments, a quadrupole time-of-flight (Q-TOF) instrument (maXis 4G; Bruker, Bremen, Germany) and an ion trap instrument (amaZon speed ETD; Bruker, Bremen, Germany). Standard source settings (capillary voltage 4.5 kV, nebulizer gas pressure 0.5 bar, drying gas 5 L/min, 200 °C) were used. Instrument tuning was optimized for a mid mass range (500–3000-Da molecules).

The purified samples were loaded on a PGC column (100 mm x 0.32 mm, 5 μm; Thermo Scientific, Waltham, MA, USA) with use of 65 mM ammonium formate buffer of pH 3.0 (positive mode) or 10 mM ammonium carbonate (negative mode) as the aqueous solvent. A gradient from 1% solvent B (80% acetonitrile plus 20% solvent A) to 68% solvent B in 40 min was applied, at a flow rate of 6 μL/min. Detection was performed with the Q-TOF or ion trap mass spectrometer equipped with the standard ESI source in data-dependent acquisition mode (switching to MS/MS mode for eluted peaks), directly linked to the Thermo Ultimate 3000 UPLC system. MS scans were recorded (range 150–2200 Da for the Q-TOF instrument and 400–1600 Da for the ion trap instrument) and the four highest peaks were selected for fragmentation. Instrument calibration was performed with an ESI calibration mixture (Agilent).

Data interpretation was done with DataAnalysis 4.0 (Bruker, Bremen, Germany). Theoretical isotopic distributions were calculated with Compass isotope pattern calculator (Bruker, Bremen, Germany). For quantification with the “all peaks” strategy, the four (Man5) to seven (G4S4F) highest isotopic peaks (resulting in at least 96.5% of the overall theoretical peak areas) in the extracted ion chromatogram were integrated (detailed in Table S1). All charge states and adduct ions were considered if their top peak reached at least 4% of the base peak of the particular spectrum.

## Results

### MS spectra of glycans in positive and negative mode

**Fig. 1:**
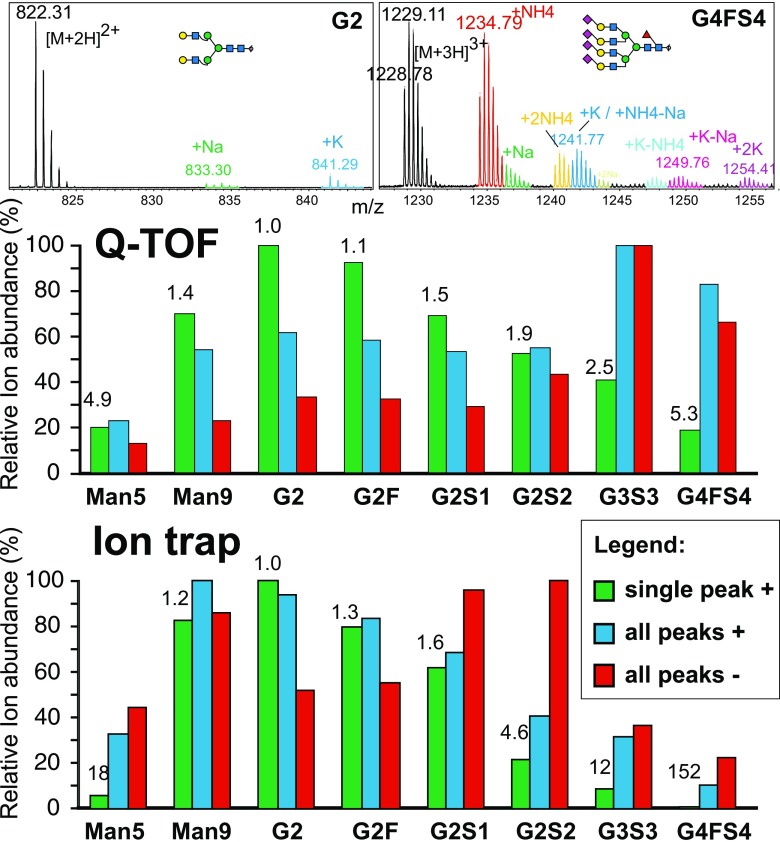
Peak fine structures and ion abundancies of mass spectrometry (MS) peaks as determined with the equimolar “glyco tune mix.” The mixture was subjected to porous graphitic carbon liquid chromatography (PGC-LC) with MS detection in either positive mode or negative mode with a maXis 4G quadrupole time-of-flight (*Q-TOF*) instrument or an amaZon ion trap instrument (both Bruker). The *top panels* show the isotope and adduct footprints of a smaller, neutral and a large tetrasialylated N-glycan. The *lower panels* compare the ion abundances as determined from extracted ion chromatograms for either only the monoisotopic peak (“single peak”) or “all” peaks (see “Materials and methods”). The *numbers above the bars* are the correction factors for the “single peak” approach relative to G2. Technical error was below 3%

When preparing the glycans for the equimolar reference mixture (Table 1), we stumbled on the startling complexity of MS results for glycans. First, this arises from the splitting of peaks into isotope patterns, which differ depending on the size of the glycans (Fig. 1). Very different results are thus obtained when the monoisotopic peak, the most abundant peak, or “all” peaks (covering 96.5% of the theoretical isotope pattern; all charge states and adduct peaks) were considered. In addition, most *N*-glycans tend to occur in two charge states in ESI and form adduct peaks of different relative height (Fig. 1). The most relevant adduct peaks in PGC LC–ESI-MS in positive mode correspond to ammonium-containing ions as the eluents usually used contain either ammonium formate (for positive mode analysis [32, 33]) or ammonium carbonate (for negative mode analysis [3]). In negative mode, carbonate adducts were generated, whereas formate adducts were found if the eluent contained ammonium formate (not shown). Some complica-tion arises from the oligomannosidic glycans’ tendency to undergo in-source fragmentation, which is almost absent for Man9 but increases from Man8 to Man5.

### A “glyco tune mix” for correction of ion abundances and its application to the plasma glycome

Ion abundances (intensities) can be obtained from either the monoisotopic peaks or the entire footprint of the analyte. In the absence of an appropriate standard, a makeshift solution toward relative quantification could be to consider all isotopic peaks (more exactly, those making up at least 96.5% of the entire theoretical isotope cluster as detailed in Table S1), charge states, and adducts of an analyte. This approach, however, resulted in ion abundance values highly discrepant with the actual stoichiometry (Fig. 1). The most serious deviations were observed between neutral and highly sialylated species, and here again with the ion trap instrument in positive mode (blue bars in Fig. 1), where the apparent ratio between G4FS4 and neutral G2 was 1:9.3 despite their being present in equal molar amounts. The smallest divergence was seen again with the ion trap instrument in negative mode, with a spread of 1:4.5 (red bars in Fig. 1) between G4FS4 and the disialylated G2S2. So, in addition to the enormous differences in molar response, even the relative order of the glycans differed between polarities and instruments. The values actually measured are highly dependent on the tuning of the instrument and thus cannot be transferred from one instrument to another as was already obvious from the again rather different relative responses previously found with a Waters Q-TOF instrument in negative mode [32]. The inferior performance of the ion trap instrument for highly sialylated glycans may reflect inadequate tuning, but it also reminds us of the poor sensitivity found for sialylated glycopeptides when the same type of trap was used [42].

Uncorrected quantification via only the monoisotopic peak of the most relevant ion type yielded even more distorted results, with a spread of 20 and 150 for the Q-TOF instrument and the ion trap instrument respectively. However, use of the results obtained with the equimolar standard allows the calculation of correction factors, which would compensate for different responses. Then again, one has the choice of using the analyte’s entire footprint (isotope peaks, charges states, adducts) or just the monoisotopic peak of the most abundant charge state. The “all peaks” strategy—though preferable if no correction is applied—has three obvious disadvantages: (1) it requires more work to the set up the extracted ion chromatogram parameters, (2) it may require more time for extraction of the respective traces, and, most relevant, (3) it maximizes the risk of collecting peaks from contaminants. Although this risk also exists if only the monoisotopic peak is considered, it can largely be avoided—or at least noticed—by verification of the isotopic distribution of this one ion type. Although in this study we used monoisotopic masses throughout for the “single peak” strategy, choosing the second isotope peak could be a worthwhile choice for larger glycans. Notably, the ion abundance values for smaller glycans such as Man5 are unduly low because of their tendency to fragment.

**Fig. 2:**
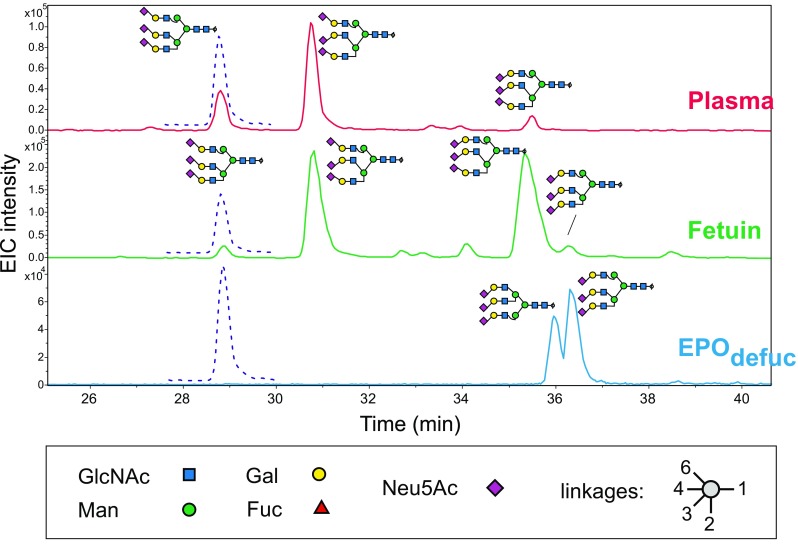
PGC-LC separation of triantennary, trisialylated *N*-glycans in the presence of the isotope-coded standard ^C13^Na^6-4^[Na^6-4^Na^6-4^]. Extracted ion chromatograms for *m*/*z* = 961.68 (*solid lines*) and *m*/*z* = 963.68 (*dashed lines*) are shown. Peak annotations of serum and fetuin glycans are based on previous structural elucidation [37] together with the empirical rule that α-2,3-linked sialic acids cause increased retention. Sialic acids (*diamonds*) pointing upward are α-2,6-linked, those pointing downward α-2,3-linked. *EIC* extracted ion chromatogram*, EPO* erythropoietin, *Fuc* fucose, *Gal* galactose, *GlcNAc N*-acetylglucosamine, *Man* mannose, *Neu5Ac N*-acetylneuraminic acid

Plasma contains many more structures than just the seven contained in the standard mixture [8, 43]. Some of these additional glycans are just isomers with, for example*,* different sialic linkages or branching (Fig. 2), and it may well be assumed that these have similar response factors within the boundaries of measuring accuracy (typically around 5%) (Table 2). Other structures differ, for example, by the presence/absence of fucose, galactose, or a bisecting GlcNAc, and the deviations may be larger, but correction with factors for the respective “nearest neighbor” will yield reasonably good approximations of true values (Table 2). Large differences in retention time and hence solvent composition may reduce the gain in correction. As an example, the acetonitrile content changes by a factor of 1.25 during the elution of the trisialoglycans (Fig. 2) and thus may lead to deviations that, however, appear small compared with the huge original error.

**Table 2:** Abundance of *N*-glycans in human serum. Relative molar abundances were calculated from the peak areas of the monoisotopic peaks and normalized to the earliest eluted G2S2 (Na^6-4^Na^6-4^) variant by the respective correction factors given in Fig. 1

	Isomer	Uncorrected relative abundance (%)		Instrument discrepancy before correction	Corrected relative abundance (%)		Instrument discrepancy after correction	Concentration (pmol/μL)
		Q-TOF	Ion trap		Q-TOF	Ion trap		
	1	0.94 ± 0.06	2.7	2.8	0.51	0.65	1.3	1.1
G2F	1	6.8 ± 0.2	14.5	2.1	4.5	4.6	1.0	8.9
G2S1	1	26.5 ± 0.9	54.5	2.1	20.2	19.1	1.1	38.4
	2	1.6 ± 0.1	2.5	1.6	1.2	0.89	1.4	2.1
	3	0.9 ± 0.1	ND		0.7	ND		1.3
G2S2	1	100	100	1.0	100	100	1.0	195
	2	12.5 ± 0.2	13.7	1.1	12.5	13.7	1.1	25.6
G3S3	1	3.8 ± 0.2	2.8	1.5	4.9	7.3	1.5	12.0
	2	10.1 ± 0.3	4.5	2.2	13.0	11.7	1.1	24.1
	3	1.56 ± 0.03	ND		2	ND		3.9
G0F	1	8.3 ± 0.6	22.1	2.7	4.4	4.8	1.1	8.9
G1F^a^	1	15.6 ± 0.4	39.3	2.5	8.2	8.5	1.0	16.3
G2FS1^a^	1	6.6 ± 0.1	11.4	1.7	5.7	5.9	1.0	11.4
G2FS2^a^	1	9.5 ± 0.1	6.9	1.4	10.7	10.0	1.1	20.3

Absolute quantification was performed with ^C13^G2S2 as an internal standard with the quadrupole time-of-flight (*Q-TOF*) instrument

*ND* not determined

^a^Not included in equimolar mix. Correction was performed with the “nearest neighbor” rule; for example, G1F values were corrected with the factor for G1.

The equimolar “glyco tune mix,” whose concentration is known, could be applied for absolute quantitation via external calibration, and this strategy has just recently been realized by others [35]. For work with complex samples such as plasma or tissues, which require extensive processing steps before the actual analysis, it appears advisable to take into account losses during workup. Hence, we decided to generate stable-isotope-labeled standards, as detailed in the following section.

### Preparation of ^13^*C*_6_-galactose-labeled oligosaccharides

**Fig. 3:**
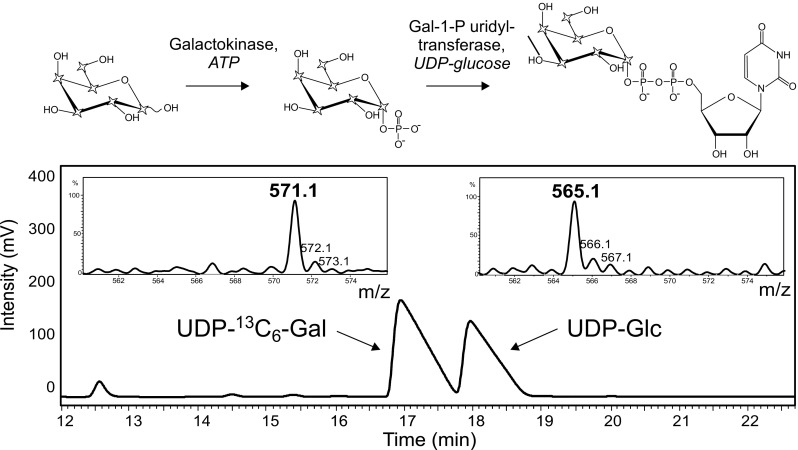
Preparation and isolation by PGC-LC of UDP-^13^ *C*_6_-galactose. Isolated peaks were subjected to ion trap MS. *Stars* symbolize ^13^C atoms in the chemical formulas. *Gal* galactose, *Gal-1-P* galactose 1-phosphate, *Glc* glucose

We used the Leloir pathway for the generation of UDP-^13^ *C*-galactose [44]. ^13^ *C*_6_-galactose is phosphorylated and—in the same pot—transferred to UDP in exchange for unlabeled glucose (Fig. 3). Usually, more than half of the UDP-glucose is converted to UDP-galactose. Attempts at optimizing the yield were not made [45]. The desired product can be easily discriminated by mass from unlabeled UDP-glucose. The two nucleotide sugars in the reaction mixture were separated by PGC chromatography (Fig. 3) even though a perfect purification is not required as neither bovine β-1,4-galactosyltransferase nor human β-1,3-galactosyltransferase used UDP-glucose (data not shown).

GnGn (i.e., a desialylated and degalactosylated diantennary *N*-glycan) was incubated with UDP-^13^ *C*_6_-galactose and β-1,4-galactosyltransferase. The fully galactosylated product ^C13^A^4^A^4^ (or ^C13^G2) was separated from the partially galactosylated isomers ^C13^A^4^Gn and ^C13^GnA^4^ by PGC-HPLC on a column with an inner diameter of 3 mm. The ^C13^G2 fraction was further treated with ST6Gal to arrive at singly sialylated (^C13^G2S1; actually a mixture of Na^6-4^A^4^ and A^4^Na^6-4^) and doubly sialylated (^C13^G2S2 or exactly ^ic^Na^6-4^Na^6-4^, ic standing for isotope coded) glycans, which were isolated by PGC-LC and thus the charged species ^C13^G2 and ^C13^G2S2 (and ^C13^G2S1) were available in pure form and could be individually quantitated via amino sugar analysis.

Preparation of structures with more antennae and more sialic acids was undertaken by the stripping off of sialic acids and—partially—galactose from fetuin glycans. The fraction with two galactose residues was isolated by HPLC and furnished with one ^13^ *C*_6_-galactose to arrive at ^C13^G3S3 (exactly ^C13^Na^6-4^[Na^6-4^Na^6-4^] in ProGlycAn nomenclature (http://www.proglycan.com). Complete regalactosylation with ^13^ *C*_6_-galactose would have shifted the mass into a region already occupied by various adduct ions (Fig. 1), hence the choice of only one ^13^ *C*_6_-galactose. Though successful, this route all too obviously was not suitable for a routine, large-scale preparation. Therefore, the synthesis of triantennary and tetraantennary glycans was postponed in exchange for the combination of a broad-range nonlabeled standard set with just one or two isotope-coded standards for absolute quantification. However, for the interpretation of elution patterns of plasma, fetuin, or erythropoietin glycans the isotope-coded trisialoglycan proved useful (Fig. 2).

**Fig. 4:**
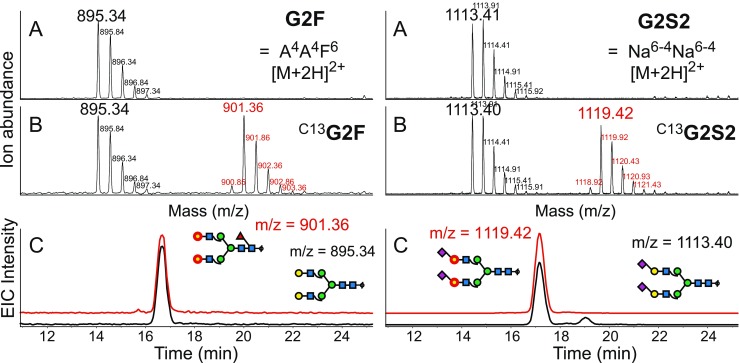
Mass spectra of G2 and G2S2 in unspiked and spiked samples (*A*, *B*). Note that the isotope-coded glycans emerge in unoccupied spaces in the spectra. The spectra demonstrate the different isotope distributions of natural glycans and 12 ^13^C-containing glycans. *Panels C* confirm the identical chromatographic behavior of the isotopologues. The ^13^ *C*_6_-galactose residues are labeled with *red circles. EIC* extracted ion chromatogram

The isotope-coded standards ^C13^G2 and ^C13^G2S2 fortunately occupy parts of the mass spectrum where no adduct ions interfere (Fig. 4). A small complication occurs when the isotope-labeled standards are used with the one-peak method as the isotope pattern of the labeled glycan is not only shifted by 12 Da but is also altered because of the imperfect isotopic purity of the ^13^C-labeled galactose (Fig. 4). The supplier stated 1% ^12^C, but inspection of the UDP-galactose suggested only 0.85% impurity. This impurity results in a theoretical error of 8.21%; that is, the area of the monoisotopic peak of ^C13^G2 (^C13^A^4^A^4^) should be multiplied by 1.089 to allow a theoretically correct comparison with natural G2 (A^4^A^4^).

### Absolute quantification of human plasma glycans and the mysterious glycosylation of bovine serum albumin

The validity of the approach was tested with human transferrin. With two relevant N-glycosylation sites mainly occupied by G2S2 (i.e., Na^6-4^Na^6-4^) [46], 70 μg (about 1 nmol) of transferrin is expected to contain about 1.7 nmol G2S2. The experimental result gave a content of 1.23 nmol per nanomole (data not shown). The difference may in part arise from the moisture content of the glycoprotein.

Applying the same method to human plasma resulted in absolute concentrations of plasma *N*-glycans (Table 2). Whereas immunglobulins are a very abundant class of glycoproteins in serum, their Fc glycans (G0F, G1F, G2F, and G2FS1) do not dominate the glycan profile.

A curious case emerged recently when several (glyco)proteins were analyzed by NMR spectroscopy. HSA gave clear signals for disialo diantennary *N*-glycans (G2S2) [47]. As HSA is generally not considered to be N-glycosylated, this surprise may come from rare alleles or from impurities [48, 49]. To determine this, we quantitated the amount of a possible glycoprotein. We found 67 μg (1 nmol) HSA contained 0.15 nmol G2S2, mainly in the form of Na^6-4^Na^6-4^. Thus, a glycosylated albumin allele would amount to about 15%. However, a tryptic digest revealed substantial amounts of serotransferrin, haptoglobin, hemopexin, and α-1B-glycoprotein (MASCOT scores 892, 712, 382, and 64; HSA itself was identified with a MASCOT score of 3503) as contaminants, and it appears justifiable to regard these glycoproteins as the source of the NaNa seen by NMR spectroscopy. These (mostly smaller) glycoproteins bear two or more complex *N*-glycans and thus may amount to 4-7% of the material. A glycosylated HSA variant of about 15% would also be seen by intact protein ESI-MS [50], but no such variant was found by this approach (data not shown).

## Discussion

External calibration using a set of *N*-glycans of known—preferably equal—concentrations appears to be the minimum requirement for quantitative evaluation of glycomics data obtained by ESI-MS of underivatized glycans. Without this correction, the data are hardly more than incidental peak height ratios that will in addition vary from day to day and instrument to instrument. A recent attempt at external calibration showed impressive linearity over several orders of magnitude of permethylated glycans for a Thermo Fisher LTQ Orbitrap instrument [35]. Two nongalactosylated, neutral glycans (GnGnbi and Gn[GnGn]bi in ProGlycAn nomenclature) and one disialoglycan (Na^6-4^Na^6-4^) were the subjects of this study. A larger panel of structures was used in a study that introduced the application of stable-isotope-labeled glycans for internal calibration [30]. This work focused on IgG glycans and thus on diantennary structures with zero to two galactoses and fucose and up to two sialic acids. However, as IgG glycans were analyzed by MALDI-TOF MS, no further attention was paid to sialylated species.

In this work, we considered a broader range of *N*-glycans from Man5 to tetrasialylated *N*-glycan (Fig. 1, Table 1). These standards were prepared as reduced glycans suitable for PGC-LC–ESI-MS, a method that is suitable for both neutral and sialylated glycans and that has an unsurpassed ability to separate isomeric forms (e.g., different Neu5Ac linkage [33] or fucose linkage). The equimolar glyco tune mix can be used to optimize the instrument tuning for glycan analysis and it can be used to measure the different molar responses. By application of the correction factors obtained to *N*-glycans of identical or very similar composition, meaningful abundance values for essentially all glycan species can be obtained.

Without correction factors, acceptable ratios were obtained for complex glycans with zero to two sialic acids with the Q-TOF MS system (Fig. 1, Table 2). However, this may only hold true for this particular instrument and its tuning. In the absence of any verification by a standard mixture, even these common glycan species may give deviating ion abundance responses.

Absolute quantitation could—in principle—be obtained by external calibration. We noticed, however, that sample preparation for LC–ESI-MS resulted in considerable loss (about 50%) of material. This problem can be overcome by the addition of an internal standard at the earliest possible time during sample processing (e.g., at the end of the enzymatic deglycosylation). This standard must differ from the sample (i.e., it must be stable isotope labeled). In the present work we accomplished this task by a route that exclusively uses the tools of a biochemistry laboratory. In fact, all enzymes required can be obtained commercially. We assume, however, that such isotope-labeled standards will find their most relevant application not in absolute quantitation but rather in isomer assignment by PGC-LC–ESI-MS as shown in Fig. 2. Much of the diversity of the triantennary glycans shown in this example arises from different sialic acid linkages, which could also be determined via differential derivatization and MALDI-TOF MS [8]. The branching pattern of erythropoietin glycans and many other isomer details can, however, be revealed only by LC–MS.

The isotope labeling of *N*-glycans with three and more antennae becomes increasingly difficult and economically questionable. Hence, we suggest use of a “broad-range” nonlabeled standard for instrument tuning and relative quantitation. If desired, absolute quantitation can then easily be achieved with just one or possibly two isotope-labeled glycans (e.g., G2 and G2S2) as demonstrated with human plasma and impurities of HSA.

This two-stage strategy can also be realized with the help of isotope-labeled standards prepared by a different route as offered by Asparia Glycomics (https://aspariaglycomics.com/). A unique advantage of the ^13^C-galactose approach is the possibility of generating asymmetrically labeled compounds, where just one arm has received the heavy form, as such compounds allow the inspection of the fragmentation behavior under various conditions (data not shown). Another unique option offered by the enzymatic approach is isotope coding of glycopeptides or whole glycoproteins for isotope dilution or pulse-chase experiments.

Taken together, our findings demonstrate the need to consider the differing molar responses of sugars of different composition, size, and sialic acid content. The suggested approach is external calibration with an equimolar glycan mixture that has already displayed its usefulness during instrument tuning. Absolute quantitation by the addition of an isotope-labeled internal standard can then be performed if required.

## Electronic supplementary material

Below is the link to the electronic supplementary material.

ESM 1 (PDF 153 kb)
