# Supplementary material for: Determination of true ratios of different N-glycan structures in electrospray ionization mass spectrometry
Source: Anal Bioanal Chem. 2017 Mar 7;409(10):2519–30. doi: 10.1007/s00216-017-0235-8 (PMC5359385; doi:10.1007/s00216-017-0235-8)
Supplement: Supplementary file 1 — (PDF 153 kb) [file 216_2017_235_MOESM1_ESM.pdf]

Analytical and Bioanalytical Chemistry

Electronic Supplementary Material

Determination of true ratios of different N-glycan structures in electrospray ionization-mass spectrometry

Clemens Grünwald-Gruber, Andreas Thader , Daniel Maresch, Thomas Dalik, Friedrich Altmann

Table S1 Glycan structures, their masses composition and contribution to total peak area

Part A - Structures and their composition

			Number of Monosaccherides											
#	structure	Mass [M+H]$^+$	HexNAc	Hex	Pent	dHex	NeuAc	Hex*C	C	H	O	N	S	^{13}C
1	G4FS4	3684.31954	6	7	0	1	4	0	140	230	102	10	0	0
2	G3S3	2882.03404	5	6	0	0	3	0	109	180	80	8	0	0
3	G2S2	2225.80644	4	5	0	0	2	0	84	140	62	6	0	0
4	G2S1	1934.71104	4	5	0	0	1	0	73	123	54	5	0	0
5	Man5	1237.45684	2	5	0	0	0	0	46	80	36	2	0	0
6	G2	1643.61564	4	5	0	0	0	0	62	106	46	4	0	0
7	G2F	1789.67354	4	5	0	1	0	0	68	116	50	4	0	0
8	^{13C}G2S2	2237.84674	4	3	0	0	2	2	72	140	62	6	0	12
9	Man9	1885.66804	2	9	0	0	0	0	70	120	56	2	0	0

Part B - Theoretical isotopic patterns

					topic distribution (highest peak 100%) (calculated with Isotope pattern								
#		Chemical formula	-2	-1	*Mono-isotopic*	*1st*	*2nd*	*3rd*	*4th*	*5th*	*6th*	*7th*	*8th*
1	G4FS4	H230 C140 N10 O102 S0 *C0			61.864	100	93.22	63.59	35.02	16.35	6.664	2.422	0.759
2	G3S3	H180 C109 N8 O80 S0 *C0			79.388	100	75.48	42.18	19.21	7.449	2.474	0.658	
3	G2S2	H140 C84 N6 O62 S0 *C0			100	97.04	59.3	27.08	10.18	3.269	0.825		
4	G2S1	H123 C73 N5 O54 S0 *C0			100	84.27	46.15	18.94	6.435	1.871	0.43		
5	Man5	H80 C46 N2 O36 S0 *C0			100	52.78	21.05	6.11	1.51	0.302			
6	G2	H106 C62 N4 O46 S0 *C0			100	71.52	34.65	12.58	3.811	0.893			
7	G2F	H116 C68 N4 O50 S0 *C0			100	78.28	40.49	15.71	5.058	1.395	0.305		
8	^{13C}G2S2	H140 C72 N6 O62 S0 *C12	0.45	9.82	100	81.8	45.7	19.3	6.79	2.04	0.49		
9	Man9	H120 C70 N2 O56 S0 *C0			100	79.97	43.07	17.39	5.851	1.686	0.384		

Part C – Calculation of minimal number of peaks for the 96.5 % treshold

		Number of isotopic peaks:			
#		*4th*	*5th*	*6th*	*7th*
	**Structure**	% of total peak volume when considering 4-7 peaks - cut off 96.5%			
1	G4FS4	83.89	93.11	**97.41**	99.16
2	G3S3	90.89	**96.76**	99.04	99.80
3	G2S2	95.21	**98.62**	99.72	
4	G2S1	**96.62**	99.11	99.83	
5	Man5	**97.89**	99.60		
6	G2	**97.20**	98.35		
7	G2F	**99.00**	99.83		
8	^{13C}G2S2	96.33	98.88	! here the frame is shifted to the left [illegible]	
9	Man9	**96.81**	99.17		

Part D – masses and m/z values for the components of the glyco tune-mix

				masses used for EIC generation are shown in red					
**Glycoform**	**Adduct**	**Mass [M+H]+**	**Charge**	**mono-isotopic**	**1st isotope peak**	**2nd**	**3rd**	**4th**	**5th**
UU*	H+	589.245153	2	295.1226	295.6262	296.1298	296.6335	297.1371	297.6408
MU*	H+	751.297953	2	376.1490	376.6526	377.1562	377.6599	378.1635	378.6672
MM*	H+	913.350753	2	457.1754	457.6790	458.1826	458.6863	459.1899	459.6936
Man4*	H+	1075.403553	2	538.2018	538.7054	539.2090	539.7127	540.2163	540.7200
*… in source fragmentation of Man5 observed. Fragmentation products were considered									
Man5	H+	1237.456353	1	1237.4564	1238.4636	1239.4709	1240.4782	1241.4854	1242.4927
Man5	NH4	1254.482353	1	1254.4824	1255.4896	1256.4969	1257.5042	1258.5114	1259.5187
Man5	Na	1259.438851	1	1259.4389	1260.4461	1261.4534	1262.4607	1263.4679	1264.4752
Man5	H+	1237.456353	2	619.2282	619.7318	620.2354	620.7391	621.2427	621.7464
Man5	NH4	1254.482353	2	627.7412	628.2448	628.7484	629.2521	629.7557	630.2594
Man5	Na	1259.438851	2	630.2194	630.7231	631.2267	631.7303	632.2340	632.7376
Man5	K	1275.41279	2	638.2064	638.7100	639.2137	639.7173	640.2209	640.7246
G2S1	H+	1934.710515	2	967.8553	968.3589	968.8625	969.3662	969.8698	970.3734
G2S1	H+	1934.710515	3	645.5702	645.9059	646.2417	646.5774	646.9132	647.2490
G2S1	Na	1956.693013	2	978.8465	979.3501	979.8538	980.3574	980.8610	981.3647
G2S1	K	1972.666952	2	986.8335	987.3371	987.8407	988.3444	988.8480	989.3517
G2S2	H+	2225.805932	2	1113.4030	1113.9066	1114.4102	1114.9139	1115.4175	1115.9211
G2S2	H+	2225.805932	3	742.6020	742.9377	743.2735	743.6092	743.9450	744.2808
G2S2	Na	2247.78843	2	1124.3942	1124.8979	1125.4015	1125.9051	1126.4088	1126.9124
G2S2	NH+	2242.831932	2	1121.9160	1122.4196	1122.9232	1123.4269	1123.9305	1124.4341
G2S2	K+	2263.762369	2	1132.3812	1132.8848	1133.3885	1133.8921	1134.3957	1134.8994
G2S2	Na	2247.78843	3	749.9295	750.2652	750.6010	750.9367	751.2725	751.6083
G2S2	K+	2263.762369	3	755.2541	755.5899	755.9256	756.2614	756.5971	756.9329
G3S3	H+	2882.033544	2	1441.5168	1442.0204	1442.5240	1443.0277	1443.5313	1444.0349
G3S3	H+	2882.033544	3	961.3445	961.6803	962.0160	962.3518	962.6875	963.0233
G3S3	NH4+	2899.059544	2	1450.0298	1450.5334	1451.0370	1451.5407	1452.0443	1452.5479
G3S3	NH4+	2899.026	3	967.0087	967.3444	967.6802	968.0159	968.3517	968.6875
G3S3	Na+	2904.016042	2	1452.5080	1453.0117	1453.5153	1454.0189	1454.5226	1455.0262
G3S3	Na+	2904.016042	3	968.6720	969.0078	969.3435	969.6793	970.0150	970.3508
G3S3	K+	2919.989981	2	1460.4950	1460.9986	1461.5023	1462.0059	1462.5095	1463.0132
G3S3	K+	2919.989981	3	973.9967	974.3324	974.6682	975.0039	975.3397	975.6754
G4FS4	H+	3684.319065	2	1842.6595	1843.1632	1843.6668	1844.1704	1844.6741	1845.1777
G4FS4	H+	3684.319065	3	1228.7730	1229.1088	1229.4445	1229.7803	1230.1160	1230.4518
G4FS4	NH4+	3701.345065	2	1851.1725	1851.6762	1852.1798	1852.6834	1853.1871	1853.6907
G4FS4	NH4+	3701.345065	3	1234.4484	1234.7841	1235.1199	1235.4556	1235.7914	1236.1271
G4FS4	Na+	3706.301563	3	1236.1005	1236.4363	1236.7720	1237.1078	1237.4435	1237.7793
G4FS4	K+	3722.275502	3	1241.4252	1241.7609	1242.0967	1242.4324	1242.7682	1243.1040
G4FS4	2NH4	3718.371065	3	1240.1237	1240.4594	1240.7952	1241.1310	1241.4667	1241.8025
G4FS4	K+NH4	3739.301502	3	1247.1005	1247.4363	1247.7720	1248.1078	1248.4435	1248.7793
G2 (AA)	H+	1643.6151	2	822.3076	822.8112	823.3148	823.8185	824.3221	824.8257
G2 (AA)	Na+	1665.597598	2	833.2988	833.8024	834.3061	834.8097	835.3133	835.8170
G2 (AA)	K+	1681.571537	2	841.2858	841.7894	842.2930	842.7967	843.3003	843.8039
G2F (AAF)	H+	1789.673	2	895.3365	895.8401	896.3438	896.8474	897.3510	897.8547
G2F (AAF)	NH4	1806.699	2	903.8495	904.3531	904.8568	905.3604	905.8640	906.3677
G2F (AAF)	Na+	1811.655498	2	906.3277	906.8314	907.3350	907.8387	908.3423	908.8459
G2F (AAF)	K+	1827.629437	2	914.3147	914.8184	915.3220	915.8256	916.3293	916.8329
Man9	H+	1885.66804	1	1885.6680	1886.6753	1887.6826	1888.6899	1889.6971	1890.7044
Man9	H+	1885.66804	2	943.3340	943.8377	944.3413	944.8449	945.3486	945.8522
Man9	NH4	1902.69404	2	951.8470	952.3507	952.8543	953.3579	953.8616	954.3652
Man9	Na+	1907.650538	2	954.3253	954.8289	955.3325	955.8362	956.3398	956.8434
Man9	K+	1923.624477	2	962.3122	962.8159	963.3195	963.8231	964.3268	964.8304
